# Supplementary material for: Proteotranscriptomic Profiling of the Toxic Mucus of Kulikovia alborostrata (Pilidiophora, Nemertea)
Source: Toxins (Basel). 2024 Dec 26;17(1):5. doi: 10.3390/toxins17010005 (PMC11768458; doi:10.3390/toxins17010005)
Supplement: Supplementary file 1 [file toxins-17-00005-s001.zip › Supplementary File S4.zip!/ORF 076246 LINALB/5646dc0998f06131/~ORF076246.html]

Phyre 2 Results for putative\_toxin\_cistein\_rich\_


|  |  |
| --- | --- |
|  | Retrieve Phyre Job Id |


|  |  |
| --- | --- |
| Email | vas9i-kz@mail.ru |
| Description | putative\_toxin\_cistein\_rich\_ |
| Date | Wed Apr 5 07:02:50 BST 2023 |
| Unique Job ID | 5646dc0998f06131 |
| Sequence | KRCNPKGSFC ... Download FASTA |
| Job Type | **normal** |
| Job Expiry |  |

|  |  |
| --- | --- |
| Summary |  |

Top model

Image coloured by rainbow N → C terminus

|  |  |
| --- | --- |
| Model dimensions (Å): | **X**:17.906 **Y**:21.043 **Z**:19.080 |

|  |  |
| --- | --- |
|  |  |
 Close JSmol | | |

 Model (left) based on template c6enaA\_ Top template information **PDB header:**toxin **Chain:** A: **PDB Molecule:**nemertide alpha-1; **PDBTitle:** nemertide alpha-1 **PDB Entry:** PDBe RCSB PDBj - Confidence and coverage Confidence: \| **94.4%** \| Coverage: \| **81%** 25 residues ( 81% of your sequence) have been modelled with 94.4% confidence by the single highest scoring template. 3D viewing Interactive 3D view in JSmol For other options to view your downloaded structure offline see the FAQ |

|  |  |
| --- | --- |
| Sequence analysis |  |

|  |  |
| --- | --- |
|  | Download FASTA version |

|  |  |
| --- | --- |
| Secondary structure and disorder prediction [Show] |  |

|  |  |  |  |
| --- | --- | --- | --- |
|  |  |  |  |

|  |  |  |  |  |  |  |  |  |  |  |  |  |  |  |  |  |  |  |  |  |  |  |  |  |  |  |  |  |  |  |  |  |  |  |  |  |  |  |  |  |  |  |  |  |  |  |  |  |  |  |  |  |  |  |  |  |  |  |  |  |  |  |  |  |  |  |  |  |  |  |  |  |  |  |  |  |  |  |  |  |
| --- | --- | --- | --- | --- | --- | --- | --- | --- | --- | --- | --- | --- | --- | --- | --- | --- | --- | --- | --- | --- | --- | --- | --- | --- | --- | --- | --- | --- | --- | --- | --- | --- | --- | --- | --- | --- | --- | --- | --- | --- | --- | --- | --- | --- | --- | --- | --- | --- | --- | --- | --- | --- | --- | --- | --- | --- | --- | --- | --- | --- | --- | --- | --- | --- | --- | --- | --- | --- | --- | --- | --- | --- | --- | --- | --- | --- | --- | --- | --- | --- |
|  |  | 1 | . | . | . | . | . | . | . | . | 10 | . | . | . | . | . | . | . | . | . | 20 | . | . | . | . | . | . | . | . | . | 30 | . |  |  |  |  |  |  |  |  |  |  |  |  |  |  |  |  |  |  |  |  |  |  |  |  |  |  |  |  |  |  |  |  |  |  |  |  |  |  |  |  |  |  |  |  |  |  |  |  |
| Sequence |  | K | R | C | N | P | K | G | S | F | C | T | K | N | N | N | C | C | N | Y | P | P | K | C | S | W | A | F | K | C | T | S |  |  |  |  |  |  |  |  |  |  |  |  |  |  |  |  |  |  |  |  |  |  |  |  |  |  |  |  |  |  |  |  |  |  |  |  |  |  |  |  |  |  |  |  |  |  |  |  |
| Secondary structure |  | --- | --- | --- | --- | --- | --- | --- | --- |  |  |  | --- | --- | --- |  |  |  | --- | --- | --- | --- | --- | --- | --- |  |  |  |  |  |  | --- |  |  |  |  |  |  |  |  |  |  |  |  |  |  |  |  |  |  |  |  |  |  |  |  |  |  |  |  |  |  |  |  |  |  |  |  |  |  |  |  |  |  |  |  |  |  |  |  |
| SS confidence |  | --- | --- | --- | --- | --- | --- | --- | --- | --- | --- | --- | --- | --- | --- | --- | --- | --- | --- | --- | --- | --- | --- | --- | --- | --- | --- | --- | --- | --- | --- | --- |  |  |  |  |  |  |  |  |  |  |  |  |  |  |  |  |  |  |  |  |  |  |  |  |  |  |  |  |  |  |  |  |  |  |  |  |  |  |  |  |  |  |  |  |  |  |  |  |
| Disorder |  | **?** | **?** | **?** | **?** | **?** | **?** | **?** | **?** | **?** | **?** | **?** | **?** | **?** | **?** | **?** | **?** | **?** | **?** | **?** | **?** | **?** | **?** | **?** | **?** | **?** | **?** | **?** | **?** | **?** | **?** | **?** |  |  |  |  |  |  |  |  |  |  |  |  |  |  |  |  |  |  |  |  |  |  |  |  |  |  |  |  |  |  |  |  |  |  |  |  |  |  |  |  |  |  |  |  |  |  |  |  |
| Disorder confidence |  | --- | --- | --- | --- | --- | --- | --- | --- | --- | --- | --- | --- | --- | --- | --- | --- | --- | --- | --- | --- | --- | --- | --- | --- | --- | --- | --- | --- | --- | --- | --- |  |  |  |  |  |  |  |  |  |  |  |  |  |  |  |  |  |  |  |  |  |  |  |  |  |  |  |  |  |  |  |  |  |  |  |  |  |  |  |  |  |  |  |  |  |  |  |  |
|  | | | | | | | | | | | | | | | | | | | | | | | | | | | | | | | | | | | | | | | | | | | | | | | | | | | | | | | | | | | | | | | | | | | | | | | | | | | | | | | | |

|  |  |  |  |  |  |  |  |  |  |  |  |
| --- | --- | --- | --- | --- | --- | --- | --- | --- | --- | --- | --- |
| Confidence Key | | | | | | | | | | | |
| High(9) |  |  |  |  |  |  |  |  |  |  | Low (0) |

|  |  |
| --- | --- |
| **?** | Disordered ( 29%) |
|  | Alpha helix ( 0%) |
|  |  |

Beta strand ( 39%)

|  |  |
| --- | --- |
| Domain analysis [Show] |  |

|  |  |  |  |
| --- | --- | --- | --- |
|  |  |  |  |

|  |
| --- |
| (Rank \| Aligned region) |
| (1 \| (--- \| c6enaA\_ \| ---); PDB 6ena chain A **Region:** 3 - 29 **Aligned:** 25 **Modelled:** 27 **Confidence:** 94.4% **Identity:** 52% **PDB header:**toxin **Chain:** A: **PDB Molecule:**nemertide alpha-1; **PDBTitle:** nemertide alpha-1 **PDB Entry:** PDBe RCSB PDBj Phyre2; 2 \| (--- \| d1h5oa\_ \| ---); PDB 1h5o chain A **Region:** 1 - 29 **Aligned:** 29 **Modelled:** 29 **Confidence:** 22.4% **Identity:** 38% **Fold:** Defensin-like **Superfamily:** Defensin-like **Family:** Myotoxin Phyre2; 3 \| (--- \| c1z99A\_ \| ---); PDB 1z99 chain A **Region:** 1 - 29 **Aligned:** 29 **Modelled:** 28 **Confidence:** 18.4% **Identity:** 38% **PDB header:**toxin **Chain:** A: **PDB Molecule:**crotamine; **PDBTitle:** solution structure of crotamine, a myotoxin from crotalus durissus2 terrificus **PDB Entry:** PDBe RCSB PDBj Phyre2; 4 \| (--- \| d1vtxa\_ \| ---); PDB 1vtx chain A **Region:** 3 - 27 **Aligned:** 24 **Modelled:** 25 **Confidence:** 44.8% **Identity:** 42% **Fold:** Knottins (small inhibitors, toxins, lectins) **Superfamily:** omega toxin-like **Family:** Spider toxins Phyre2) |

|  |  |
| --- | --- |
| Detailed template information [Hide] |  |

|  |  |  |  |
| --- | --- | --- | --- |
|  |  |  |  |

| # | Template | Alignment Coverage | 3D Model | Confidence | % i.d. | Template Information |
| --- | --- | --- | --- | --- | --- | --- |

|  |  |  |  |  |  |  |
| --- | --- | --- | --- | --- | --- | --- |
| 1 | c6enaA\_ | --- \| --- \| --- |  | 94.4 | 52 |


 (**PDB header:**toxin **Chain:** A: **PDB Molecule:**nemertide alpha-1; **PDBTitle:** nemertide alpha-1 **PDB Entry:** PDBe RCSB PDBj) |
| 2 | d1h5oa\_ | --- \| --- \| --- |  | 22.4 | 38 | **Fold:** Defensin-like **Superfamily:** Defensin-like **Family:** Myotoxin **PDB entry:** PDBe RCSB PDBj |
| 3 | c1z99A\_ | --- \| --- \| --- |  | 18.4 | 38 |


 (**PDB header:**toxin **Chain:** A: **PDB Molecule:**crotamine; **PDBTitle:** solution structure of crotamine, a myotoxin from crotalus durissus2 terrificus **PDB Entry:** PDBe RCSB PDBj) |
| 4 | d1vtxa\_ | --- \| --- \| --- |  | 44.8 | 42 | **Fold:** Knottins (small inhibitors, toxins, lectins) **Superfamily:** omega toxin-like **Family:** Spider toxins **PDB entry:** PDBe RCSB PDBj |


|  |  |
| --- | --- |
| Binding site prediction |  |

Automated 3DLigandsite submission is temporarily suspended due to server load. However you may submit models directly to 3DLigandSite HERE

---

Phyre is now FREE for commercial users!

All images and data generated by Phyre2 are free to use in any
publication with acknowledgement

**Please cite:** The Phyre2 web portal for protein modeling, prediction and analysis.

Kelley LA *et al.*. *Nature Protocols*
10, 845-858 (2015)[pdf]
[Citation
link]

If you use the binding site
predictions from 3DLigandSite, **please also cite:**

3DLigandSite: predicting ligand-binding sites using similar structures.

Wass MN, Kelley LA and Sternberg
MJ *Nucleic Acids Research* 38, W469-73 (2010) [PubMed]

© Structural Bioinformatics Group

Imperial College London

Lawrence
Kelley, Michael Sternberg

Disclaimer

Terms and Conditions

|  |
| --- |
| **Component software** |
| Template detection: HHpred 1.51 |
| Secondary structure prediction: Psi-pred 2.5 |
| Disorder prediction: Disopred 2.4 |
| Transmembrane prediction: Memsat\_SVM |
| Multi-template modelling and *ab initio*: Poing 1.0 |
